# Protective Role of Combined Polyphenols and Micronutrients against Influenza A Virus and SARS-CoV-2 Infection In Vitro

**DOI:** 10.3390/biomedicines9111721

**Published:** 2021-11-19

**Authors:** Marta De Angelis, David Della-Morte, Gabriele Buttinelli, Angela Di Martino, Francesca Pacifici, Paola Checconi, Luigina Ambrosio, Paola Stefanelli, Anna Teresa Palamara, Enrico Garaci, Camillo Ricordi, Lucia Nencioni

**Affiliations:** 1 Laboratory Affiliated to Istituto Pasteur-Fondazione Cenci Bolognetti, Department of Public Health and Infectious Diseases, Sapienza University of Rome, 00185 Rome, Italy; marta.deangelis@uniroma1.it (M.D.A.); Annateresa.palamara@iss.it (A.T.P.); 2 Department of Systems Medicine, University of Rome “Tor Vergata”, 00133 Rome, Italy; francesca.pacifici@uniroma2.it; 3 Department of Human Sciences and Quality of Life Promotion, San Raffaele Roma Open University, IRCCS San Raffaele Roma, 00166 Rome, Italy; paola.checconi@uniroma5.it (P.C.); enrico.garaci@sanraffaele.it (E.G.); 4 Department of Neurology and Evelyn F. McKnight Brain Institute, Miller School of Medicine, University of Miami, Miami, FL 33136, USA; 5 Department of Infectious Diseases, Istituto Superiore di Sanità, 00161 Rome, Italy; gabriele.buttinelli@iss.it (G.B.); angela.dimartino@iss.it (A.D.M.); luigina.ambrosio@iss.it (L.A.); paola.stefanelli@iss.it (P.S.); 6 Cell Transplant Center, Diabetes Research Institute, University of Miami Miller School of Medicine, Miami, FL 33136, USA; ricordi@miami.edu

**Keywords:** SARS-CoV-2, influenza virus, antivirals, polyphenols, micronutrients, COVID-19, polydatin

## Abstract

Polyphenols have been widely studied for their antiviral effect against respiratory virus infections. Among these, resveratrol (RV) has been demonstrated to inhibit influenza virus replication and more recently, it has been tested together with pterostilbene against severe acute respiratory syndrome coronavirus 2 (SARS-CoV-2) infection. In the present work, we evaluated the antiviral activity of polydatin, an RV precursor, and a mixture of polyphenols and other micronutrients, named A5+, against influenza virus and SARS-CoV-2 infections. To this end, we infected Vero E6 cells and analyzed the replication of both respiratory viruses in terms of viral proteins synthesis and viral titration. We demonstrated that A5+ showed a higher efficacy in inhibiting both influenza virus and SARS-CoV-2 infections compared to polydatin treatment alone. Indeed, post infection treatment significantly decreased viral proteins expression and viral release, probably by interfering with any step of virus replicative cycle. Intriguingly, A5+ treatment strongly reduced IL-6 cytokine production in influenza virus-infected cells, suggesting its potential anti-inflammatory properties during the infection. Overall, these results demonstrate the synergic and innovative antiviral efficacy of A5+ mixture, although further studies are needed to clarify the mechanisms underlying its inhibitory effect.

## 1. Introduction

Since human origins, viral and microbial infections have been the most feared and dangerous killers, as recently confirmed by the coronavirus disease 2019 (COVID-19) pandemic. The severe acute respiratory syndrome coronavirus 2 (SARS-CoV2) pandemic has now caused close to 5 million deaths worldwide (www.who.int, accessed on 10 October 2021). Although different types of vaccines are now available against SARS-CoV2 and a strong campaign of vaccination is led by almost all countries in the world, a great number of people are still dying from this infection and its complications [1]. In association to vaccines, therapeutic approaches also exist. In particular, remdesivir is an antiviral agent which has been reported to reduce SARS-CoV-2 replication in both in vitro and ex vivo models [2]. Tocilizumab is a monoclonal antibody against both soluble and membrane-bound Interlukin 6 receptor (IL-6R) that significantly reduces the inflammatory response induced by COVID-19. Hydroxychloroquine (HCQ) seems to act as a viral inhibitor in in vitro studies; however, retrospective analyses conducted in COVID-19 patients were not able to demonstrate an HCQ protective effect. However, as a result of viral evolution, SARS-CoV-2 variants have spread widely and have displayed evidence for being more transmissible, causing more severe disease and/or reducing neutralization by antibodies generated during previous infection or vaccination [3]. Thus, the need for additional strategies to fight COVID-19 is evident.

Polyphenols are bioactive compounds derived from plants and produced as secondary metabolites [4]. Besides their established antioxidant and anti-inflammatory properties, several studies demonstrated their significant protection against viruses and several pathogens, including the Epstein–Barr and herpes simplex viruses [5,6,7]. We have already demonstrated the ability of resveratrol in inhibiting the replication of influenza A virus [8], and recent research in this field demonstrates that various types of polyphenols, such as resveratrol and pterostilbene, are promising antiviral compounds to inhibit SARS-CoV-2 infection [9]; indeed, other research aligns perfectly with these results [10,11].

The rationale behind this research is even stronger, since the results obtained from the computational analysis studies such as Excalate4CoV (https://www.exscalate4cov.eu/, accessed on 10 October 2021) and others [12] reported the active interface between the SARS-CoV-2 and phenolic molecular structure.

Based on these findings in the present study, we aimed to test, for the first time, a novel and patented mixture of polyphenols, identified as A5+ in in vitro models of infection by influenza A virus or SARS-CoV-2. A5+ is a mixture composed of ellagic acid, polydatin, pterostilbene and honokiol, mixed with zinc, selenium, and chromium. Polydatin and pterostilbene are natural precursors of resveratrol that have been demonstrated having stronger antioxidant and anti-inflammatory effects, and most importantly, a better bioavailability compared to resveratrol [13]. Their protective effects on COVID-19, especially those from polydatin, have recently raised great interest [14]. Ellagic acid, manly derived from pomegranate (*Punica granatum* L.) fruits, has already demonstrated an attenuating interaction between SARS-CoV-2 Spike glycoprotein and the human angiotensin-converting enzyme 2 (ACE2) receptor, which is the key event of virus infection [15]. Honokiol is a natural potent thrombosis inhibitor [16], and hypercoagulability is among the most important complications leading to death of COVID-19 [17]. Zinc, selenium and chromium have already demonstrated their protective efficacy against SARS-CoV-2 infection [11].

Therefore, our main objective was to test the possible protection of polydatin alone and the potential and the synergic beneficial antiviral effects of these polyphenols and other micronutrients mixed in a single compound.

## 2. Materials and Methods

### 2.1. Cell Cultures, Viral Infections

Vero E6 (African green monkey kidney cells, LGC) and MDCK (Madin-Darby canine kidney cells) were purchased from American Type Culture Collection (ATCC, Manassas, VI, USA) and were grown in Minimum Essential Medium (MEM) (Sigma Aldrich, St. Louis, MO, USA) supplemented with 10% fetal calf serum (FCS) (Corning, NY, USA); glutamine 0.3 mg/mL; penicillin 100 U/mL and streptomycin 100 mg/Ml (Sigma Aldrich, St. Louis, MO, USA). Confluent monolayer of cells was challenged with influenza A virus strain human A/Puerto Rico/8/34 H1N1 (PR8) for 1 h at 37°. After the viral adsorption, the cells were washed with phosphate-buffered saline (PBS) and then incubated with medium supplemented with 2% FCS for 24 h. For SARS-CoV-2 experiments, a strain isolated from a COVID-19 positive patient at the beginning of the pandemic in Italy was used [18] in the Biosafety level 3 facility of the Istituto Superiore di Sanita’ (ISS) in Rome. The virus was propagated in Vero E6 cells that were propagated at 37 °C in 5% CO_2_ in MEM supplemented with 10% FCS, 1% L-glutamine, and 1.4% sodium bicarbonate. Virus-infected cells were maintained at 37 °C in 5% CO_2_ in MEM supplemented with 2% FCS [19,20].

### 2.2. Cell Treatment

Polydatin and A5+ were provided by SirtLife srl, Rome, Italy. A5+ 40 µg is composed of ellagic acid (20%), polydatin (98%), pterostilbene (20%), honokiol (20%), mixed with recommended doses of zinc, selenium, and chromium. The polydatin and A5+ were dissolved in DMSO (Dimethyl sulfoxide, Sigma Aldrich, Milan, Italy) at 1 mg/mL.

### 2.3. Viral Titration

Hemagglutination assay: Influenza virus production was evaluated in the supernatants of infected cells recovered 24 h after treatment by measuring the hemagglutinin units (HAU). In this assay, the ability of HA molecules inserted in the envelope of mature viral particles to agglutinate erythrocytes is tested using human type 0 Rh+ erythrocytes [21]. Control conditions were reported as infected treated DMSO or H_2_O cells at the same concentration present in the test substance being evaluated.

Tissue Colture Infectious Dose 50 (TCID_50_) assay: SARS-CoV-2 titers were measured by TCID_50_ system in Vero E6 cell. Briefly, samples were serially diluted 1/10 in medium. Then, 100 µL of each dilution was plated into 10 wells of 96-well plates containing 80–90% confluent cells. The plates were incubated at 37 °C under 5% CO_2_ for five days. Each well was then scored for the presence or absence of the virus. The limiting dilution end point (CCID50/mL) was determined by the Kärber equation [22].

Plaque assay: SARS-CoV-2 titers were also determined by plaque assay in Vero E6 cells. Briefly, 6-well plates were plated with Vero E6 cells (200,000/well in MEM + 10% FCS) and, after 24 h, inoculated with 10-fold dilutions of each sample. Plates were incubated for 1 h at 37 °C, and then 3 mL/well of a medium containing 2% Gum Tragacanth (Sigma Aldrich, St. Louis, MO, USA) + MEM +2.5% FCS was added. After 5 days at 37 °C with 5% CO_2_, titers were calculated using crystal violet dye (Sigma Aldrich, St. Louis, MO, USA) in plaque forming units per milliliter [23].

Real-time PCR: The viral RNA was extracted using the Qiamp Viral RNA Mini Kit (Qiagen, Hilden, Germany), according to the manufacturer’s recommendations. SARS-CoV-2 RNA were analyzed for N2 gene by in-house rt-Real-time PCR through the Applied Biosystems 7500 Fast System instrument, using reagents and protocol from CDC (Division of Viral Diseases, Centers for Disease Control and Prevention-USA) [24].

### 2.4. Cytotoxicity Assay

The cytotoxicity assay was evaluated on Vero E6 cells used for influenza virus infection by the MTT [3-(4,5-dimethylthiazol-2-yl)-2,5-diphenyltetrazolium bromide] assay (Sigma Aldrich, St. Louis, MO, USA). Briefly, cells were seeded in 96-well plates at a density of 2 × 10^4^ cells/well in 100 μL of complete MEM medium without phenol red for 24 h at 37 °C. Subsequently, cell monolayers were treated or not with increasing concentrations (5–100 μg/mL) of Polydatin or A5+ compounds, dissolved, respectively, in H_2_O or DMSO, for the following 24 h. MTT solution (10 μL) was added to each well for 3 h at 37 °C. Then, each sample was acidified by adding 0.1 N HCl in isopropanol (100 μL/well) for 30 min to ensure that all formazan crystals were dissolved. Absorbance of samples was read at 570 nm, using an automatic plate reader (Multiskan EX, Ascent Software, Thermo Fisher Scientific, Milan, Italy). Cells treated with DMSO or H_2_O solvent were used as control.

### 2.5. In Cell Western Assay

MDCK cells were grown in 96-well plates (2 × 10^4^ cells/well), either infected or mock-infected (Ctr) with supernatants of infected and treated Vero E6 cells. After 24 h, they were fixed with 4% paraformaldehyde (Santa Cruz Dallas, TX, USA), and permeabilized with 0.1% Triton X-100 and incubated with Odyssey Blocking buffer (LI-COR Biosciences, Lincoln, NE, USA). Cells were then stained at 4 °C overnight with mouse anti-HA or anti-NP (Santa Cruz Biotechnology, Santa Cruz, CA, USA) in 5% Odyssey Blocking Buffer. Cells were then washed and stained with a mixture of fluorochrome-conjugated secondary antibodies (fluorescence emission at 800 nm) together with Cell Tag (LI-COR Biosciences, Lincoln, NE, USA) (LI-COR Biosciences, Lincoln, NE, USA) properly diluted in Odyssey Blocking buffer for 1 h at room temperature. Cell Tag was used as a control of the integrity of the cell monolayer. Subsequently, three washes with PBS (Sigma Aldrich, St. Louis, MO, USA) plus 0.1% Tween 20 were performed, and plates were analyzed by the Odyssey Imaging System (LI-COR, Lincoln, NE, USA). Integrated intensities of fluorescence were determined by the LI-COR Image Studio software and the relative fluorescence unit (RFU) was expressed as a percentage compared to untreated infected cells (100%) [25].

### 2.6. IL-6 Quantification

Cytokine quantification was measured by using IL-6 ELISA kit (Cusabio, Wuhan China). Briefly, supernatants obtained from Vero E6 infected with influenza virus A/PR/8/H1N1 and treated with A5+ (20–30 μg/mL) were added onto a microplate pre-coated with IL-6 specific antibody and any IL-6 present is bound. After removing supernatants, a biotin-conjugated antibody specific for IL-6 was added to the wells. After washing, avidin conjugated horseradish peroxidase (HRP) was added to the wells. Following a wash to remove any unbound avidin-enzyme reagent, a substrate solution was added to the wells and color develops in proportion to the amount of IL-6 bound in the initial step. The color development was stopped, and the intensity of the color was measured by a Multiskan EX Reader (Thermo Fisher Scientific, Monza, Italy).

### 2.7. Protein Extraction and Western Blot Analysis

Vero E6 infected with SARS-CoV-2 treated or not with polydatin and A5+, were lysed in RIPA buffer [20 mM Tris–HCl pH 8, 150 mM NaCl, 1% Triton X-100, 0.5% sodium dodecyl sulfate (SDS) and 1% sodium deoxycholate] supplemented with phenylmethylsulphonyl fluoride, protease inhibitor mixture, and phosphatase inhibitor (Sigma Aldrich, St. Louis, MO, USA). Subsequently, cell lysates were centrifuged (13,000 rpm, 30 min, 4 °C) and the supernatants were diluted in sodium dodecyl sulfate (SDS) sample buffer containing DL-Dithiothreitol (DTT 0.1 M) (Sigma Aldrich, St. Louis, MO, USA). The total extract was analyzed by SDS-PAGE followed by western blotting. The membranes were blocked with 10% non-fat dry milk in Tris-buffered saline containing 0.01% Tween-100 for 1 h at room temperature (RT). Primary antibodies, used at final concentration of 1 μg/mL, included mouse monoclonal anti-spike antibody (S) (Gene Tex cat No. GTX632604), rabbit polyclonal anti-nucleocapsid antibody (N) (Rockland code: 200-401-A50) and mouse monoclonal anti-actin (Sigma Aldrich, Saint Louis, MO, USA). Bound antibodies were detected using horseradish peroxidase-conjugated secondary antibodies (Jackson, Cambridge, UK) followed by Clarity Western ECL substrate (Bio-Rad, Milan, Italy).

### 2.8. Statistical Analysis

Statistical analyses were carried out using a two-tailed Student’s test. A *p* value of <0.05 was considered statistically significant. The data represents the mean of replicate experiments and the relative standard deviation (SD). Statistical analysis was performed by GraphPad PrismTM 6.0 software (GraphPad Software Inc., San Diego, CA, USA).

## 3. Results

### 3.1. Polydatin and A5+ Are Toxic on Vero E6 Cells Only at Higher Concentrations

**Figure 1:**
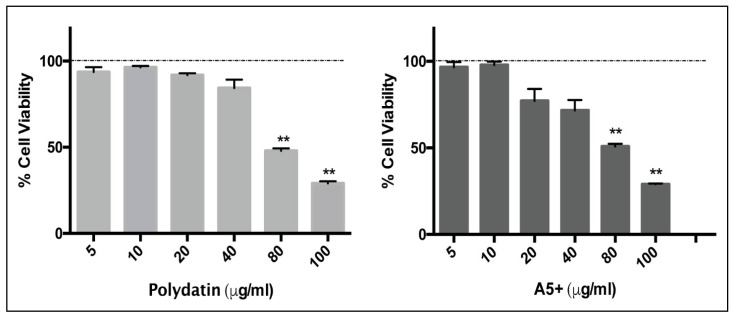
Polydatin and A5+ are toxic on Vero E6 cells only at higher concentrations. MTT assay on Vero E6 cell monolayer incubated with increasing concentrations (5–100 µg/mL) of Polydatin or A5+ compounds for 24 h. Cell viability was expressed as percentage (%) of treated cells compared to control cells treated with solvent dilutions (H_2_O or DMSO for Polydatin and for A5+ treatment, respectively). Data are mean ± SD from two technical replicates of one experiment of the two performed (** *p* < 0.001 vs. control solvent-treated cells, considered 100%, dashed line).

In order to study the antiviral activity of Polydatin and A5+ compounds against influenza virus or SARS-CoV-2, firstly the range of concentrations without cytotoxic effects on cell monolayers was established. To this aim, Vero E6 cell line was treated with different concentrations (5 to 100 µg/mL) of polydatin or A5+ and results were analyzed after 24 h of treatment. The MTT assay (Figure 1) revealed absence of any cytotoxic effect up to 40 µg/mL, which was then selected as the maximum concentration of compounds used for the analysis of its antiviral activity. In detail, the percentage of cell viability in cells treated with polydatin or A5+ (40 µg/mL) was 84 ± 3% and 71 ± 4%, respectively. A marked reduction of cell viability was found at 80 and 100 µg/mL, and these concentrations were excluded for the following experiments.

### 3.2. A5+ Compound Inhibits Influenza A Virus Replication

**Figure 2:**
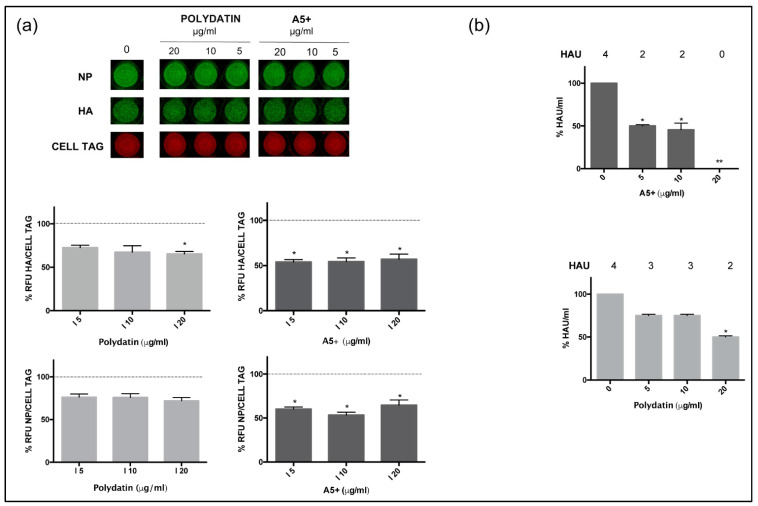
A5+ compound inhibits influenza A virus replication. (**a**) In Cell Western assay (ICW) of Hemagglutinin (HA) and Nucleoprotein (NP) expression in infected cells treated with different concentrations of polydatin or A5+ (5–20 µg/mL) for 24 h. As control, infected cells were treated with H_2_0 or DMSO at the same conditions and reported as 0 µg/mL. The green fluorescence is representative of viral proteins expression and red fluorescence of Cell Tag staining of cell monolayer. In the graphs below is reported the percentage (%) of relative fluorescence units (RFU) of HA or NP proteins expression normalized to Cell Tag and compared to control which is indicated by the dashed black line (considered as 100%). Data are expressed as means ± S.D., obtained from two independent experiments, each performed in duplicate (* *p* < 0.05 vs. infected solvent-treated cells). (**b**) Viral titer measured in the supernatants of infected treated cells (0 to 20 µg/mL of Polydatin or A5+) by Hemagglutination assay (HAU). In the graphs are represented the percentage (%) of HAU/mL compared to infected untreated condition. Data are the mean ± S.D. of two separate experiments (* *p* < 0.05 and ** *p* < 0.001 vs. Infected untreated cells, I).

Several pieces of evidence reported the antiviral efficacy of polyphenols, included resveratrol and its derivatives [26]. Our group demonstrated their ability to interfere with specific steps during the replicative cycle [8,27]. Based on this observation, we evaluated the potential anti-influenza virus activity of polydatin and A5+. Both stilbene derivative compounds (concentration range 5–20 µg/mL) were added after infection and maintained for the following 24 h. Then, supernatants from treated or not infected cells were recovered and used to infect a fresh cell monolayer of MDCK cells. As shown in Figure 2A, after 24 h infection, the immunofluorescence of two viral proteins, hemagglutinin (HA) and Nucleoprotein (NP), was quantified by In Cell Western (ICW) assay as percentage of relative fluorescence units (RFU) compared to untreated infected cells, i.e., cells treated with H_2_0 or DMSO at the same conditions of polydatin or A5+, respectively. The results showed that both polydatin and A5+ inhibited the expression of viral proteins. However, polydatin alone (20 µg/mL) reduced expression of NP by 28 ± 2%, and of HA by 35 ± 2% (* *p* < 0.05), while A5+ demonstrated a higher antiviral efficacy in both viral proteins inhibition, reaching 43 ± 4% of fluorescence reduction (* *p* < 0.05) (Figure 2a, graphs below images).

Next, the same supernatants used in the ICW assay were analyzed for viral titration by using hemagglutination (HAU) assay. Both compounds were significantly effective; nevertheless, A5+ treatment (20 µg/mL) drastically reduced the viral titer compared to DMSO-treated conditions, reporting values as solvent dilution control levels (0 µg/mL) (Figure 2b). These data confirmed the potential antiviral effect of A5+ mixture.

### 3.3. A5+ Compound Reduces IL-6 Production in Influenza A Virus-Infected Cells

Since A5+ showed a higher efficacy in inhibiting influenza virus replication compared to treatment with polydatin alone, its potential anti-inflammatory activity was further assessed by measuring one of the most important cytokines that mediates the viral inflammation, the interleukin-6 (IL-6).

**Figure 3:**
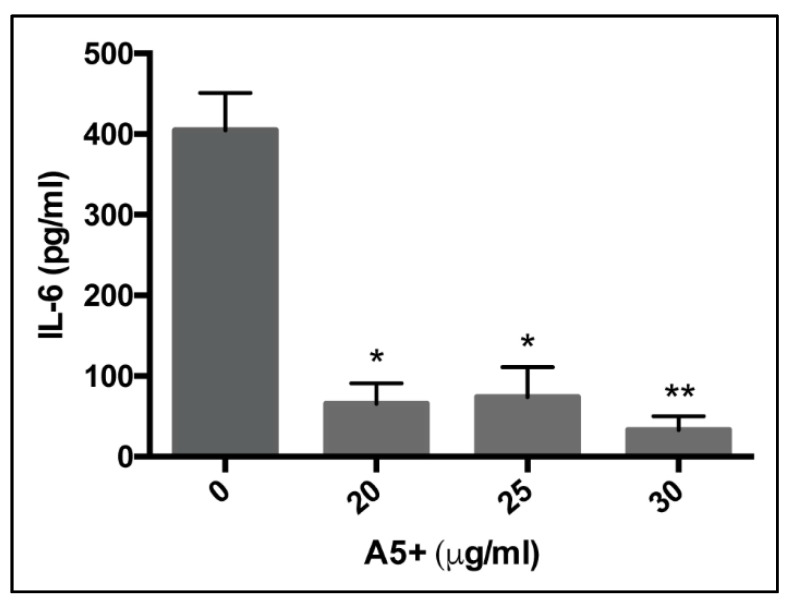
A5+ compound reduces IL-6 production in influenza virus-infected cells. IL-6 production (pg/mL) in supernatants of Vero E6 cells infected with influenza virus A/PR/8/H1N1 and treated with A5+ compound at different concentrations (20–30 µg/mL) for 24 h. DMSO-treated infected cells were used as control (0). Cytokine production was quantified by using ELISA assay as described in Materials and Methods. Results are the mean ± S.D. obtained from two independent experiments, each performed in duplicate (* *p* < 0.05 and ** *p* < 0.001 vs. infected DMSO-treated cells).

Supernatants obtained from Vero E6 cells infected and treated for 24 h were harvested and analyzed for IL-6 cytokine production by using ELISA assay. As shown in Figure 3, the treatment with A5+ reduced in a dose-dependent manner the IL-6 production compared to DMSO-treated condition, reaching the lower level of IL-6 production when A5+ was used at 30 µg/mL. Further studies will be necessary to define the mechanisms underlying this effect. Nevertheless, the results support the anti-inflammatory properties of A5+ mixture in an influenza virus infection model.

### 3.4. A5+ Treatment Reduces SARS-CoV-2 Replication When Added after Infection

**Figure 4:**
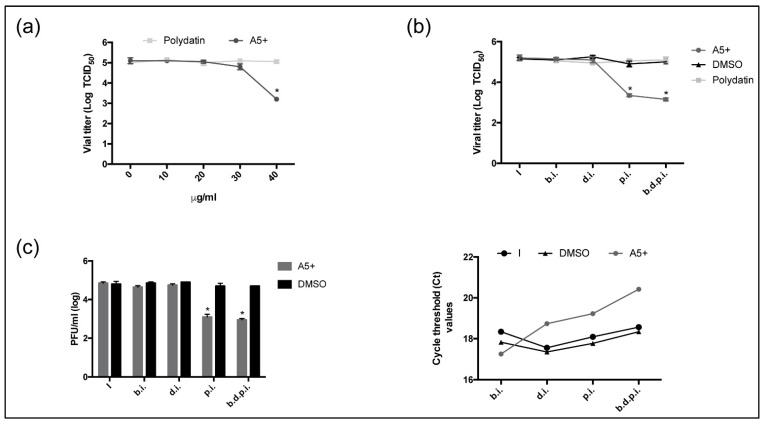
A5+ treatment reduces SARS-CoV-2 replication when added after infection. (**a**) TCID_50_ evaluated on supernatants of Vero E6 cells infected with 0.1 MOI of SARS-CoV-2 and treated with different concentrations of Polydatin or A5+ (10 to 40 μg/mL). (**b**) TCID_50_ assay on supernatants of Vero E6 infected with 0.1 MOI of SARS-CoV-2 treated with Polydatin (Poly, 40 μg/mL) or A5+ (40 μg/mL) at different steps of viral infection: cells were pretreated with compounds for 2 h at 37 °C before infection (b.i.); the compounds were added during viral challenge (d.i.) or immediately after infection (p.i.); the compounds were added overtime (before, during and post infection, b.d.p.i). DMSO was used at the same conditions in infected as control (I). (**c**) Viral titer measured in the supernatants of cells treated with A5+ (40 μg/mL) at the same conditions reported in (**b**) and expressed as plaque forming units (PFU/mL) assay (left graph) or cycles threshold (Ct) obtained by Real-Time PCR analysis (right graph). (* *p* < 0.05). TCID_50_: Tissue Culture Infectious Dose 50%; DMSO: dymethylsulfoxide; PFU: plaque forming units.

The effect of polydatin and/or A5+ was further assessed on the inhibition of SARS-CoV-2. Vero E6 cells were infected with 0.01 MOI of SARS-CoV-2 strain and both compounds (concentration range 10–40 µg/mL) were added for the following 24 h. Viral titer was evaluated in the supernatants of infected cells by Tissue Culture Infectious Dose 50% (TCID50). As shown in Figure 4A, Polydatin was not effective at any concentration tested (*p* > 0.05), while A5+ (40 µg/mL) strongly reduced viral titer (2 Logs inhibition at 40 µg/mL) compared to solvent-treated infected cells. Next, we evaluated the effect of both compounds (40 µg/mL) at different times during infection: compounds were added 2 h before infection (b.i.) on cell monolayer, during 1 h viral challenge (d.i.), or immediately post infection (p.i.); finally, the compounds were added over time (before, during and post) (b.d.p.i.) and maintained at 37 °C in a CO_2_ incubator. After 24 h, viral titer was evaluated by TCID50. As shown in Figure 4B, while polydatin did not affect viral infection at any investigated time, instead, A5+ strongly impaired viral replication when added after infection (p.i.) or when maintained over time (b.d.p.i). Indeed, values obtained in these conditions showed a significant reduction of about 1.8 and 2 Logs, respectively, compared to untreated infected cells, suggesting the most inhibition occurred during the replicative cycle of the virus.

To confirm these results, the titer in supernatant was measured by plaque forming unit (PFU/mL) assay. In these experimental conditions, we also tried a long-lasting pre-treatment (24 h) of A5+ before infection. As shown in Figure 4c (left panel), A5+ was able to reduce the viral titer in supernatant determined in plaque forming units of about 2 Logs when added post infection (p.i.) or when maintained over time (b.d.p.i), suggesting its active effect on impairment during viral replication in infected cells. Accordingly, threshold cycles (CT) measured in the supernatants of A5+ treated cells by Real-Time PCR analysis were delayed with respect to untreated cells, confirming a lesser production of viral copies in these conditions Figure 4c (right panel).

**Figure 5:**
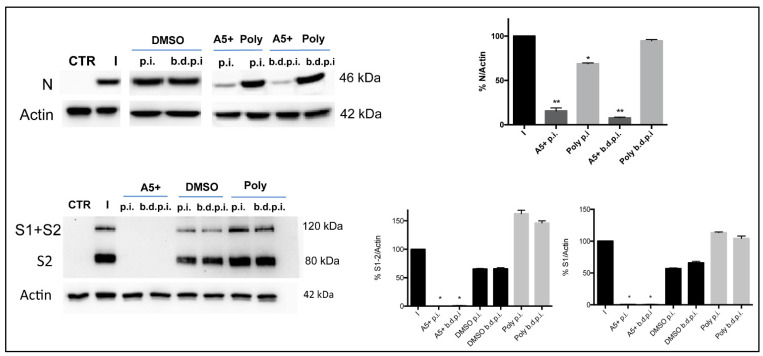
A5+ compound decreases SARS-CoV-2 proteins expression. Western blot analysis of Nucleocapsid (N) or Spike (S) proteins expression in Vero E6 cells infected with 0.1 MOI of SARS-CoV-2 and treated with 40 μg/mL of Polydatin (Poly) or A5+ for 24 h post infection (p.i.) or before, during and post infection (b.d.p.i.). DMSO was used at the same conditions in infected cells (I). Mock-infected cells were used as control of infection (CTR). Actin was used as loading control. Blot is one representative of two performed, obtained from independent experiments. The graphs represent the densitometry analysis of N or S protein expression normalized to actin and expressed as percentage (%) of treatment compared to infected cells (considered as 100%) (* *p* < 0.05 and ** *p <* 0.001 vs. I).

Finally, the analysis in western blot of SARS-CoV-2 proteins expression, Spike (S) and Nuclocapsid (N), confirmed the strong inhibition in their expression only when A5+ was added after infection. Similar results were obtained in cells treated overtime (b.d.p.i.), confirming that the prolonged pre-treatment (24 h) was quite ineffective (Figure 5).

## 4. Discussion

In the present study, we demonstrated, for the first time, the efficacy of a polyphenols’ mixture, known as A5+, against influenza A virus and SARS-CoV-2. A5+ administrated after infection significantly blunted the influenza A virus replication, measured as reduction in specific virus proteins expression, and reduction of hemagglutination reaction. This protective effect of A5+ was also shown in Vero E6 cells infected with SARS-CoV-2, where A5+ added over time and/or post infection reduced viral replication by about two Logs, even when evaluated by TCID_50_/mL than PFU/mL. These results suggest a potentially strong effect of this compound against SARS-CoV-2 infection. Moreover, we reported that treatment with A5+ caused a reduction in IL-6 levels. IL-6 production shows a key role in virus-mediated inflammation and, in particular, on COVID-19 complications [28,29]. Interestingly, A5+ revealed toxic effects only whether administrated at high concentration in vitro and a significant greater efficacy of polydatin, which is among the most powerful polyphenols against COVID-19 [30] in terms of protection against viruses’ replication.

As presented, the rationale of the present study was to test the combination of different polyphenols mixed in a unique compound along with other micronutrients against virus infection, and in particular against influenza A virus, and SARS-CoV-2. Recently, Umeoguaju et al. [31] reported, in a systematic review, how existing food plants inhibit viral pathogenesis in the human respiratory tract. The evidence suggested that plant-derived food grade substances, such as flavonoids, including ellagic acid, and resveratrol, affected different stages in the life cycle of respiratory viruses, such as influenza A virus [31]. We also demonstrated as a singular phenol, like resveratrol or its derivatives, was able to decrease viral titers in in vitro and in vivo models of influenza A virus infection [8]. Other phenols, such as quercetin or extracts from plants, have been shown to inhibit expression of viral proteins synthesis in cells infected with different strains of influenza virus [32,33], suggesting an effect on these phytocompounds in impairing viral transcription and replication activities. Furthermore, inhibition of viral NP nuclear-cytosolic traffic blocks maturation of viral particles [34]. Here, we demonstrated as polydatin alone significantly decreased expression of NP, while A5+ significantly decreased expression of both proteins, HA and NP. Polydatin is considered among the most powerful polyphenols, and it has been proved against influenza virus [35], however, we found that it is the combined and integrated activity of the compound contained into A5+ that may contribute to having the stronger effect. This was also demonstrated by the significant reduction in terms of viral titer measured by HAU after A5+ treatment compared to polydatin and DMSO.

Remarkably, A5+ also reduced production of IL-6 in cells infected with IAV, suggesting its potential effect in the reduction of inflammation and immune mediators after viral infection with different IAV viruses, such as the virus A/PR/8/H1N1. This anti-inflammatory effect can be due to several compounds present in A5+, such as zinc, selenium, and chromium [11], likewise the presence of polyphenols [11]. It has been shown, for instance, that the addition of selenium to chokeberry juice, which is rich in polyphenols, enhances its anti-inflammatory activity [36]. More recently, a herbal drug containing—among its several components—polydatin and quercetin, is already used in the Traditional Chinese Medicine for the treatment of the upper respiratory tract infections; it has been shown to have antiviral and broad anti-inflammatory effects, with a reduction of IL-6, IL-10, TNF-α, IFN-γ in coronavirus-infected mice [37]. This may be also important for the pivotal effect that IL-6 and inflammatory response play during the progression of the COVID-19 and its complications [38]; levels of IL-6 were reported significantly higher among COVID-19 patients with more severe disease or who died compared to patients with mild disease. IL-6 may be considered one of the key factors for the subsequent evolution of COVID-19 among hospitalized patients [38]. Further studies are needed to investigate the mechanisms underlying A5+ anti-inflammatory effects, aiming at fathom signaling pathways, such as PI3K/Akt and NF-kB pathways, that regulate inflammatory cytokines production.

Here, we demonstrated that A5+ added after in vitro infection, or when maintained over time, strongly impaired SARS-CoV-2 replication, viral titer in the supernatant, and viral proteins synthesis at doses that are not cytotoxic. Polydatin alone did not result protective against COVID-19 infection, further supporting the hypothesis that it has no identified mechanism proving its use against SARS-CoV-2 [39]. Conversely, A5+ works by blunting viral replication in infected cells. A recent study, conducted using the in vitro screening of drug libraries methodology, identified ellagic acid to be among the most powerful SARS-CoV-2 receptor binding domain inhibitors [40]. Pterostilbene showed a strong anti-SARS-CoV-2 effect not only in Vero E6 cells, but also in the biologically highly relevant primary human bronchial epithelial cell model when added after infection [9]. The mechanisms of this antiviral protective effect may be multiple and linked to Protein Kinase C and Sirtuin1 pathways, as already suggested [11]; indeed, Even honokiol already exhibited remarkable inhibition of SARS-CoV-2 infection in Vero E6 cells by blocking the PI3K/Akt/mTOR signaling pathway [41]. Increasing levels of selenium, and zinc in blood have been accompanied by a decrease in serum CRP level, inflammation, and severity of symptoms in COVID-19 patients, suggesting that supplementation with these micronutrients is pivotal to fight SARS-CoV-2 infection [42]. Taken together, all these findings may explain the strong effect of A5+ against SARS-CoV-2 infection. By working at different levels and with concomitant impact on virus replication mechanisms, these compounds composing A5+ may ensure a unique kind of protection.

It is remarkable that A5+ suppresses viral titer at doses that are not cytotoxic, either for IAV or for SARS-CoV-2. We must acknowledge the limitation of an in vitro study, and therefore, further experiments using other more physiological models are imperative to further demonstrate the efficacy against viruses of these compounds in humans.

## 5. Conclusions

In conclusion, in the present study, we innovatively demonstrated as a unique mixture of powerful polyphenols and micronutrients may be beneficial against influenza A virus and SARS-CoV-2 infections.

## Data Availability

The data presented in this study are available on request from the corresponding authors.
